# Complete patient exposure during paediatric brain cancer treatment for photon and proton therapy techniques including imaging procedures

**DOI:** 10.3389/fonc.2023.1222800

**Published:** 2023-09-19

**Authors:** Marijke De Saint-Hubert, Guillaume Boissonnat, Uwe Schneider, Christian Bäumer, Nico Verbeek, Johannes Esser, Jörg Wulff, Florian Stuckmann, Finja Suesselbeck, Racell Nabha, Jérémie Dabin, Fabiano Vasi, Stephan Radonic, Miguel Rodriguez, Anne Catherine Simon, Neige Journy, Beate Timmermann, Isabelle Thierry-Chef, Lorenzo Brualla

**Affiliations:** ^1^ Belgian Nuclear Research Center (SCK CEN), Mol, Belgium; ^2^ CEA, Université Paris-Saclay, Palaiseau, France; ^3^ Physik Institut, Universitat Zürich, Zürich, Switzerland; ^4^ West German Proton Therapy Centre Essen WPE, Essen, Germany; ^5^ West German Cancer Centre (WTZ), Essen, Germany; ^6^ Radiation Oncology and Imaging, German Cancer Consortium DKTK, Essen, Germany; ^7^ Department of Physics, TU Dortmund University, Dortmund, Germany; ^8^ Faculty of Mathematics and Science Institute of Physics and Medical Physics, Heinrich-Heine University, Düsseldorf, Germany; ^9^ Hospital Paitilla, Panama City, Panama; ^10^ Instituto de Investigaciones Científicas y de Alta Tecnología INDICASAT-AIP, Panama City, Panama; ^11^ INSERM U1018, Paris Sud-Paris Saclay University, Villejuif, France; ^12^ Faculty of Medicine, University of Duisburg-Essen, Essen, Germany; ^13^ Department of Particle Therapy, University Hospital Essen, Essen, Germany; ^14^ Barcelona Institute of Global Health (ISGlobal), Barcelona, Spain; ^15^ University Pompeu Fabra, Barcelona, Spain; ^16^ CIBER Epidemiología y Salud Pública, Madrid, Spain

**Keywords:** photon radiotherapy, proton therapy, out-of-field dosimetry, imaging dosimetry, Monte Carlo simulation, secondary cancer risk

## Abstract

**Background:**

In radiotherapy, especially when treating children, minimising exposure of healthy tissue can prevent the development of adverse outcomes, including second cancers. In this study we propose a validated Monte Carlo framework to evaluate the complete patient exposure during paediatric brain cancer treatment.

**Materials and methods:**

Organ doses were calculated for treatment of a diffuse midline glioma (50.4 Gy with 1.8 Gy per fraction) on a 5-year-old anthropomorphic phantom with 3D-conformal radiotherapy, intensity modulated radiotherapy (IMRT), volumetric modulated arc therapy (VMAT) and intensity modulated pencil beam scanning (PBS) proton therapy. Doses from computed tomography (CT) for planning and on-board imaging for positioning (kV-cone beam CT and X-ray imaging) accounted for the estimate of the exposure of the patient including imaging therapeutic dose. For dose calculations we used validated Monte Carlo-based tools (PRIMO, TOPAS, PENELOPE), while lifetime attributable risk (LAR) was estimated from dose-response relationships for cancer induction, proposed by Schneider et al.

**Results:**

Out-of-field organ dose equivalent data of proton therapy are lower, with doses between 0.6 mSv (testes) and 120 mSv (thyroid), when compared to photon therapy revealing the highest out-of-field doses for IMRT ranging between 43 mSv (testes) and 575 mSv (thyroid). Dose delivered by CT ranged between 0.01 mSv (testes) and 72 mSv (scapula) while a single imaging positioning ranged between 2 _μ_Sv (testes) and 1.3 mSv (thyroid) for CBCT and 0.03 _μ_Sv (testes) and 48 _μ_Sv (scapula) for X-ray. Adding imaging dose from CT and daily CBCT to the therapeutic demonstrated an important contribution of imaging to the overall radiation burden in the course of treatment, which is subsequently used to predict the LAR, for selected organs.

**Conclusion:**

The complete patient exposure during paediatric brain cancer treatment was estimated by combining the results from different Monte Carlo-based dosimetry tools, showing that proton therapy allows significant reduction of the out-of-field doses and secondary cancer risk in selected organs.

## Highlights

- Complete patient exposure during paediatric brain cancer treatment is estimated by combining different dosimetry tools.
- Imaging dose significantly contributes to the out-of-field doses in proton therapy while its contribution is proportionally much lower for photon treatments.
- Proton therapy allows to considerably decrease the out-of-field doses and thus risk of secondary cancer when compared to photon therapy.

## 1 Introduction

Improvements of radiotherapy procedures have had a major impact on survival of paediatric patients. While benefits to the patient largely outweigh risks associated with the therapeutic use of ionising radiation, the late effects of exposure are particularly important to understand for children with high probability of tumour control.

Recent large cohort studies of children exposed to low doses from computerised tomography (CT) scans have shown increased risks of leukaemia and brain tumours (1–5). Very recently, the results of the EPI-CT study, i.e. the European project on radiation-related risk of cancer in a large multinational cohort of more than one million paediatric patients involved in CT scanning, reported on a significant dose-response relationship between CT-related radiation exposure and brain cancer and emphasised careful justification of paediatric CTs and use of doses as low as reasonably possible (6). Large-scale follow-up of childhood cancer survivors has been performed for patients exposed before 2000 and for exposures to older techniques, such as 2D and early 3D conformal radiotherapy techniques (7–9). A more recent epidemiological study on the risk of a secondary cancer diagnosis showed to be similar after intensity-modulated radiotherapy (IMRT) versus 3D-conformal radiotherapy (3D-CRT), whereas proton therapy pencil beam scanning (PBS) was associated with a lower risk of secondary cancer (10). However, some epidemiological studies have failed to provide convincing evidence of the lower risk associated to proton therapy with respect to photon therapy, mainly due to small sample sizes (particularly for paediatric patients), too short follow-up times (less than 10 years for the majority of patients), and potential selection (e.g. indication, follow-up) and confounding (e.g. insufficient information on chemotherapy) biases (11, 12).

In this context, the HARMONIC project (13, 14) aims at complementing these recent studies by improving the understanding of the health effects of medical ionising radiation exposure of paediatric patients. This HORIZON 2020 European Commission project, not only addresses the issues on secondary cancer risk, but also risks associated with other late outcomes (including endocrine dysfunctions, cardio- and neurovascular damages, and patient/parent-reported quality of life, fatigue and educational outcomes) and the construction of the necessary infrastructure for their future study.

Paediatric patients undergoing radiotherapy are exposed to ionising radiation, as a consequence of the treatment, but also from complementing imaging procedures. Experimental dosimetry studies have been performed extensively within the European Radiation Dosimetry Group (EURADOS) WG9, using paediatric anthropomorphic phantoms during photon therapy (15–17) and more recently during proton therapy (18–20). Furthermore, Athar et al. (21) simulated out-of-field doses for an 8-year phantom and different 6-MV IMRT plans were compared with passive and active proton therapy techniques (21). However, only rarely were data complemented with doses from imaging (15, 22).

Until now, the imaging dose during radiotherapy was generally considered negligible in clinical practice because of its low magnitude compared to the therapeutic dose given at the treated volume. Nevertheless, the use of on-board imaging (OBI) for accurate patient positioning has become even more frequent for advanced radiotherapy, such as proton therapy. Therefore, sufficient attention should be given to the dose delivered to the patient by imaging procedures. Moreover, doses from therapeutic exposures should be complemented with imaging doses to have a complete picture of the absorbed dose distribution.

Within HARMONIC, a tool for calculating the dose from imaging procedures during radiotherapy has been further developed (23). Furthermore, HARMONIC has invested substantial efforts into validating computational and analytical tools needed to estimate out-of-field organ doses in children treated with photon and proton therapy (24, 25). Particularly important for proton therapy are the challenges related to the creation of secondary neutrons and the higher relative biological effectiveness (RBE) of neutrons and protons when compared to photons. Previous work shows the presence of different radiation types in this mixed field of stray radiation in proton therapy including variable RBE (25, 26). We believe that it is essential to combine doses from different procedures in order to make a valid comparison between proton and photon radiotherapy.

Previous studies have used the absorbed dose (27, 28) or have applied an average quality factor for neutrons to consider the RBE of neutrons (29).

A Monte Carlo study on fetal dose during brain radiotherapy considered the biological effects of neutrons by estimating the quality factor provided in ICRP Publication 60 (ICRP 1991) for proton therapy. This enabled a fair comparison between proton and photon therapy demonstrating a 10-fold reduction in the fetal dose between PBS proton therapy, and 3D-CRT (30). Others have focused only on neutron dose equivalent and, as such, have neglected the contributions from protons close to the field and gamma contributions to the out-of-field doses (21), while others have taken care of the neutron contribution to the out-of-field dose in high energy photon treatments (31). Interestingly, a recent and unique study on measurements of secondary radiation doses in child brain cancer has allowed to compare proton therapy with photon therapy (3D-CRT, IMRT and GammaKnife) (32). Our study is complementary to the study from Knezeviˇ c´ et al., but expands to cover the complete patient exposure during paediatric brain cancer treatment, including imaging. Moreover, we projected potential subsequent lifetime risks of secondary cancers following paediatric brain radiotherapy, according to a semi-mechanistic risk model proposed by Schneider et al. (33).

## 2 Materials and methods

### 2.1 Brain cancer radiotherapy techniques

Aiming to simulate a realistic treatment of a brain tumour, a clinically applied treatment plan was transferred to the conditions of the experiment. A 7-year-old female patient was selected with a diffuse midline glioma (WHO grade IV). The patient received a combined radiotherapy and chemotherapy after R3 resection. The concerned patient was enrolled in the prospective registry study ‘KiProReg’ (German Clinical Trials Register: DRKS-ID: DRKS00005363) after consent from her legal guardians. This study was approved by the local ethics committee.

**Figure 1:**
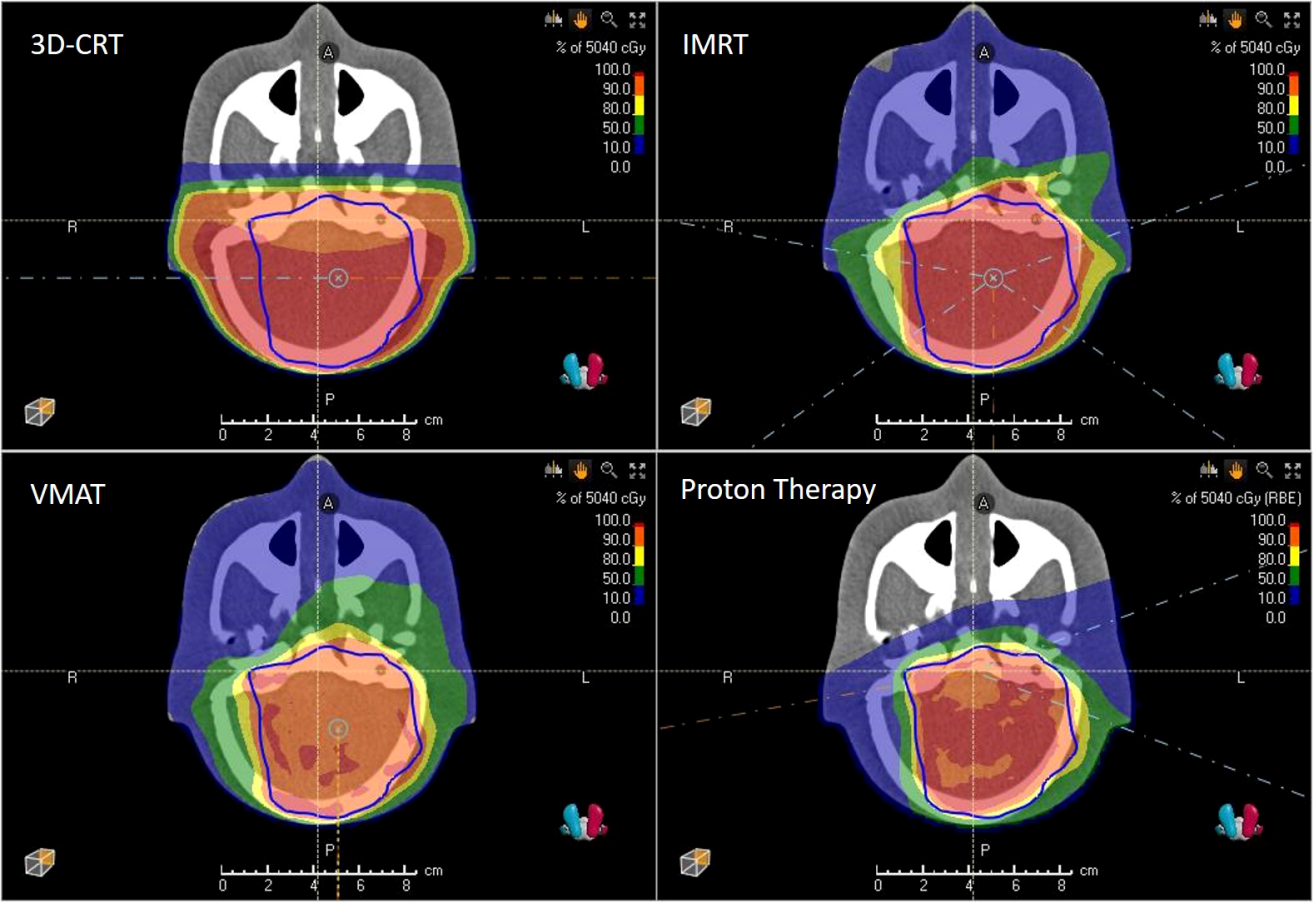
3D-CRT, IMRT, VMAT and PBS proton therapy plans showing isodoses and PTV (blue) as computed by the treatment planning system.

The clinical proton plan was transferred to an anthropomorphic phantom (ATOM, Computerized Imaging Reference Systems (CIRS), Inc.) representing a 5-year-old child (type 705D). A median dose of *D*_prescribed_ =50.4Gy(RBE) with 1.8Gy(RBE) per fraction was prescribed to the initial planning target volume (PTV), which was located in the cerebellum and had a volume of 195.2 cm^3^. The proton treatment plan consisted of two ipsilateral oblique fields and a contralateral oblique field (see **Figure 1**). The proton fields were delivered in a gantry room in PBS delivery mode employing a lucite range shifter with a thickness of 4.44cm and a water-equivalent thickness of 5.14cm. The treatment planning of the phantom case was conducted as described previously (25).

For comparison, the anthropomorphic phantom was treated with photon therapy featuring the same cranial size and shape. Three techniques were applied, namely 3D-CRT, IMRT and volumetric modulated arc therapy (VMAT). All photon irradiations for this study were done with a Varian TrueBeam STx LINAC operating with a flattening filter at a nominal energy of 6 MV. The linac was equipped with a Varian Millennium 120 multileaf collimator. The same dose of 50.4Gy with 1.8Gy per fraction was prescribed to the initial PTV. The 3D-CRT plan used two lateral fields with beam angles 90° and 270°. The IMRT plan consisted of five coplanar and isocentrical fields with beam angles of 70°, 125°, 180°, 235° and 280°, respectively. VMAT was planned using two 360° isocentric rotations. The plans were optimised with the photon optimisation algorithm PO (Varian Medical Systems, Version 13.6). The plans were iteratively optimised over several steps using the constraint V7Gy=4% for the eye and V40Gy=5% and V25Gy=5%, for the left and right cochlea respectively, and V98%[PTV]*>*95% regarding *D*_prescribed_. More details can be found in a recently published paper (24).

### 2.2 Imaging during brain cancer treatment

In order to evaluate doses delivered by X-ray based imaging systems during the course of either proton or photon therapy, Monte Carlo simulations computed the imaging absorbed dose distributions on the paediatric anthropomorphic phantom for all imaging exams that the actual treatment would have required, namely the CT exam used for planning and the OBI exams used for positioning during treatment. In practice, the proton therapy centre uses daily X-ray imaging protocol while the photon therapy centre uses daily kV-CBCT (kilo-voltage cone beam computed tomography) protocol for all radiotherapy techniques.

#### 2.2.1 Computed tomography

For planning exams, CT protocols vary very little within the same treatment centre. Nevertheless, the scan length and the X-ray tube current are often dependent on the patient morphology and pathology. Thus, we used as reference protocol the one actually delivered to the paediatric patient treated at the West German Proton Therapy Centre Essen (WPE) on its Philips Big Bore CT scanner (Philips HealthCare, The Netherlands): 120 kVp, single fixed filter, 12 mm collimation, 210 mA, 287 mm of scan length and an exposure time of 31.9 s.

#### 2.2.2 kV-CBCT

To depict the daily OBI exams performed during a radiotherapy treatment on a TrueBeam (Varian) we selected the kV-CBCT ‘head low dose’ protocol. It corresponds to an irradiation on a partial anteroposterior arc of 200° performed at 100 kVp, with the full-fan filter (with titanium foil, bowtie shaped) and 146 mAs (20 mA and 20 ms per projection and 364 projections), using 22.2 cm × 16.6 cm field size at source-axis distance (SAD). This exam is repeated at each treatment session and for the studied clinical case this corresponds to 28 times (34).

#### 2.2.3 X-ray based patient positioning and verification system

To portray the daily OBI practice, we used the WPE X-ray protocol optimised for position verification of tumours with localisation in the head of children with the proton gantry positioned at 0°. It consists of making a first image using the X-ray tube A, on the same direction as the treatment beam at 90 kVp, 12 mA and 100 ms (SAD of 1511 mm, field size of 20.2 cm × 27.9 cm); as well as a second image with X-ray tube B, oriented at 270° from the treatment beam direction at 90 kVp, 32 mA and 100 ms (SAD of 2870 mm, field size of 24.4 cm × 33.8 cm). This exam is repeated at each treatment session and for the studied clinical case, this corresponds to 28 times.

### 2.3 Monte Carlo framework

**Figure 2:**
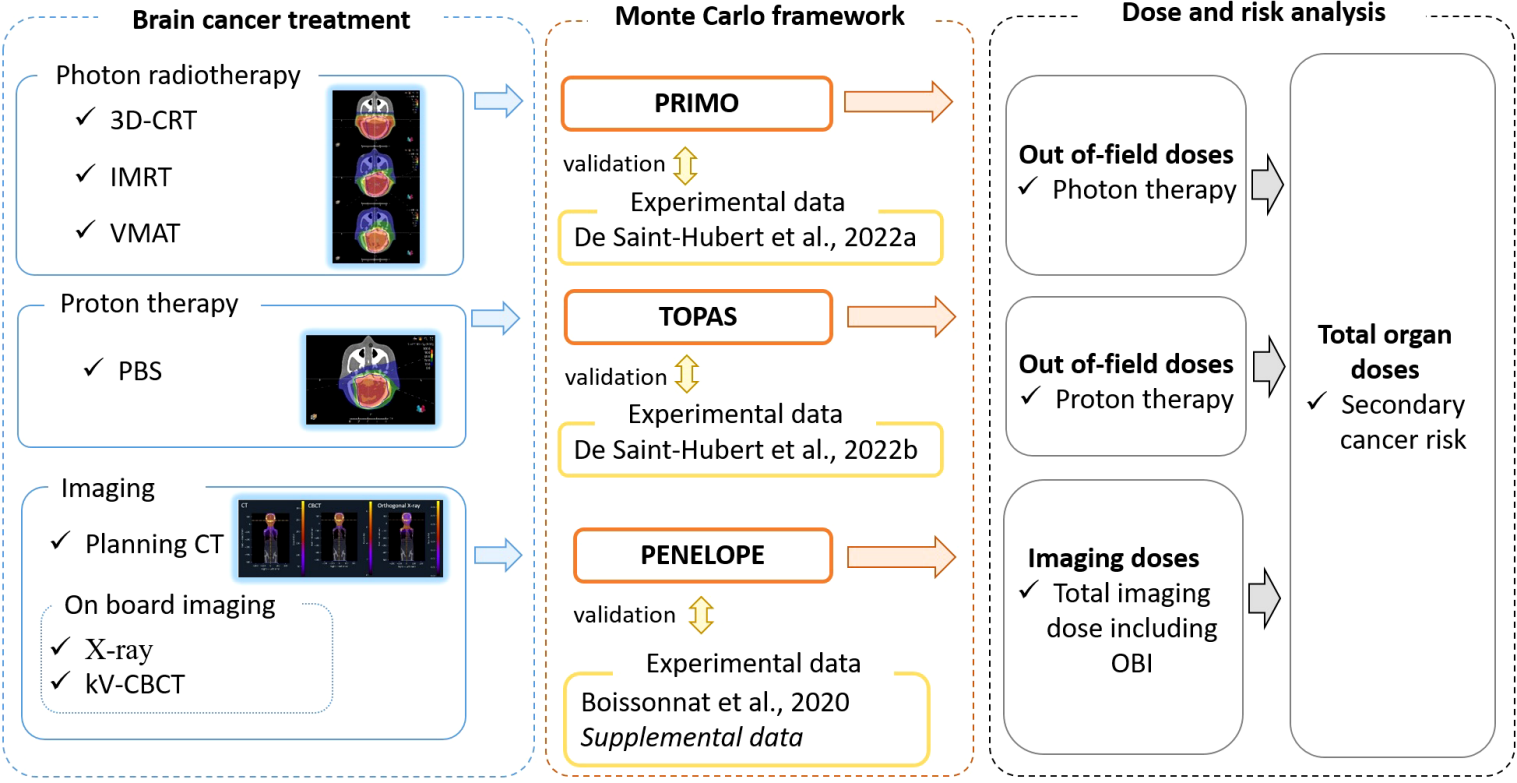
Schematic overview of the study design. Brain cancer treatment plan, involving different radiotherapy techniques and imaging protocols, were used as input to a Monte Carlo framework. This framework was validated with experimental data and provided out-of-field radiotherapy and imaging doses which were combined to derive estimates of total organ doses and secondary cancer risks.

The whole-body absorbed dose distributions presented have all been computed with general-purpose radiation transport Monte Carlo codes. In all cases, the DICOM-CT image of the anthropomorphic 5-yearold CIRS phantom was used for the Monte Carlo radiation transport. The validations of these simulations were done by comparison of the Monte Carlo-computed doses with the experimental values obtained by detectors, such as thermoluminescent detectors (TLDs) and bubble detectors, inserted in the CIRS phantom. These validations have been already published, as well as the detailed description of the simulations and experiments (24, 25). **Figure 2** shows the Monte Carlo framework used to calculate the doses from radiotherapy and imaging procedures. The Monte Carlo codes that have been used and the corresponding simulations are presented below.

#### 2.3.1 PRIMO simulations for out-of-field photon doses

PRIMO (version 1.0.64.1814) (35, 36), a Monte Carlo dose verification system that simulates medical linacs and computes the subsequent absorbed dose, was used to calculate outof-field doses in the cases of photon radiotherapy. PRIMO uses penEasy/PENELOPE (37–39) for the simulation of the radiation transport starting from the primary electron beam exiting the bending magnet, through the actual geometrical description of the linac, downstream to the collimating jaws. At that position, a phase-space was tallied, which was subsequently used as radiation source for simulating the 3D-CRT, IMRT and VMAT treatments (40). PRIMO used the fast Monte Carlo code DPM (41–43) for the simulations of these treatments and tallied the corresponding absorbed dose distributions in the CT image of the CIRS phantom. Calculated absorbed doses were converted to dose equivalent considering an RBE=/Q-factor=1. More information can be found in (24).

#### 2.3.2 TOPAS simulations for out-of-field proton doses

The Geant4 (44–46) wrap-up code TOPAS v3.6 (Geant4) (47), in conjunction with the Matlab-based (The Mathworks, Inc. Natick, Massachusetts) dose verification system matRad v2.10.1 (48), were used to simulate the out-of-field absorbed dose distribution in the case of the PBS proton therapy of the CIRS phantom. For this purpose, matRad was extended by including the possibility to process DICOM RTIon files. Thanks to this feature it was possible to create the TOPAS input files with the treatment room-specific radiation parameters. The simulations for the determination of the neutron dose equivalent at a point and the proton and gamma out-of-field dose could then be conducted. Following a validation of the Monte Carlo framework, TOPAS simulations were used to compute the total dose equivalent. Details of the experiments and simulations are given in (25).

#### 2.3.3 PENELOPE-based tool for imaging doses

The Monte Carlo framework for computing imaging absorbed doses is based on an in-house modified version of PENELOPE 2006 that introduced parallelisation and the possibility to use voxelised geometries [previously described in (23)]. Calculated absorbed doses were converted to dose equivalent considering an RBE=/Q-factor=1. This version of PENELOPE has been used previously in a software prototype dedicated to OBI dosimetry estimation as part of the Additional Imaging Doses—Image Guided Radiation Therapy project (ANR-15-CE19-0009) (49). This software already included a model of the OBI imaging system used on the TrueBeam linac and was expanded to include both the stereo imaging system used at the WPE proton beam lines and the Philips Big Bore CT scanner.

Experimental Monte Carlo model validation for both systems are presented in **Annex 1**.

### 2.4 Calculation of dose equivalent per organ

The CIRS phantom contains 180 organ-specific inserts and allowed to estimate the dose equivalent for 22 organs by averaging the calculated data from organ-specific locations. For radiotherapy we calculated the dose equivalent per organ considering a total target dose of 50.4Gy(RBE). For proton therapy, an RBE of 1.1 was considered and an absorbed dose of 45.8Gy was used for the normalization of out-of-field organ dose. For photon therapy the total absorbed target dose was 50.4Gy. For imaging, the dose equivalent per organ was calculated from a single imaging procedure for CT, kV-CBCT and X-ray. Then, we summed the dose equivalent per organ for the different imaging procedures by assuming the following: i) a single planning CT scan (1*CT) and, ii) a daily OBI (28*kV-CBCT or 28*X-ray). Finally, to get an estimate of the total dose equivalent per organ, during the entire radiotherapy treatment, the dose equivalent from radiotherapy and imaging was summed for each organ.

In the plots that follow, the error bars represent the spread on the calculated average dose equivalent per organ and not the uncertainties. The number of points in an organ varies among organs as described by the manufacturer (50). Standard statistical uncertainties of the Monte Carlo calculations are described in previous papers (24, 25), reporting up to 31% for TOPAS while for the PRIMO calculations of 3D-CRT, IMRT and VMAT the uncertainty was on average 11%. The standard statistical uncertainties of Monte Carlo calculations of the imaging procedures were on average 20%, 27%, and 16% for CT, CBCT and X-ray, respectively.

### 2.5 Lifetime attributable risk for secondary cancer

In this study we applied the carcinogenesis model, previously published (33), to estimate secondary cancer risk which emphasises cell kinetics of radiation-induced cancer by mutational processes and applies to advanced radiotherapy techniques as well as imaging dose. Briefly, the model describes carcinoma induction after fractionated radiotherapy as an analytical function and integrates cell sterilisation processes described by the linear-quadratic model and repopulation effects. The linearquadratic model of cell kill is applied to normal tissues that are irradiated during radiotherapy. Tumour induction is modelled such that each transformation process results in a tumour cell. Cancer induction in this model is a function of treatment dose, dose per fraction, defined cell kill parameters, tumour induction variable and the repopulation parameter. The obtained dose-response relationship for carcinoma induction can be used to calculate excess absolute risk (EAR):


(1) $$\text{EAR}\left(a\right)\;=\beta \left(\text{EAR}\right)\mu (e,a)\left[\frac{\exp(-\alpha^{\prime }D)}{\alpha^{\prime }R}\right]\left[1-2R+R^{2}\exp(\alpha^{\prime }D)-{\left(1-R\right)}^{2}\exp\left(-\frac{\alpha^{\prime }R}{1-R}D\right)\right]$$

The model parameters were used from the publication of Schneider et al., as obtained by fits to several epidemiological, cancer specific carcinogenesis data for carcinoma induction (33). By applying these parameters the radiation induced cancer estimates were determined. Here, *D* is the average dose equivalent, at the respective organ location, as computed within our study (units mSv) and *β*(EAR) is referring to the initial slope, which is the slope of the dose-response curve at low dose for each site. These are tabulated in Table 1 of Schneider et al. (33) for a Western population. The repopulation/repair parameter *R* characterises the repopulation/repair-ability of the tissue between two dose fractions and is 0 if no and 1 if full repopulation/repair occurs. Moreover, *α* ^′^ is the cell kill parameter for fractionated treatment as defined by:


(2) $$\alpha^{'}=\alpha +\beta \frac{D}{D_{t}}d_{t}$$

where *D*_t_ and *d*_t_ is the prescribed dose to the target volume with the corresponding fractionation dose, respectively. It is assumed here that *α/β* = 3Gy for all tissues.

The function *µ*(*e,a*) in equation 1 describes the age variation of EAR and depends on the age of exposure *e* and the attained age *a* in years:


(3) $$\mu (e,a)=\exp\left[\gamma_{e}(e-30)+\gamma_{a}\ln\left(\frac{a}{70}\right)\right]$$

The age modifying parameters *γ_e_* and *γ_a_* for a Japanese population and for different sites are taken from Table 1 in Schneider et al. (33). In this form the fit parameters are sex-averaged and centred at an age at exposure of 30 years and an attained age of 70 years. For the calculations in the present work the age of exposure was 5 years. The formulation of EAR as defined by equation 1 gives the risk of secondary cancer induction at an attained age *a*. However, it is more convenient to estimate a lifetime attributable risk (LAR) for the patient, which is the EAR integrated from *a* = *e* to the life expectancy *a*_max_. The determination of LAR was done as described by Kellerer et al. (51):


(4) $$\text{LAR }=\sum_{e}^{a_{\max}}\text{EAR(}a)\frac{S(a)}{S(e)}$$

where the survival function *S*(*a*) [taken from Kellerer et al. (51)] is the probability at birth to reach at least age *a*, while *S*(*e*) is the probability to be alive at the age of exposure. Thus *S*(*a*)*/S*(*e*) is the conditional probability of a person to be alive at age *e* and reach age *a*. LAR is calculated by summing between *e* = 5 and *a*_max_ = 90 years for six organs susceptible for secondary solid tumour induction, namely bladder, breast, liver, lung, stomach and thyroid.

## 3 Results

### 3.1 Out-of-field dose equivalent per organ during radiotherapy

**Figure 3:**
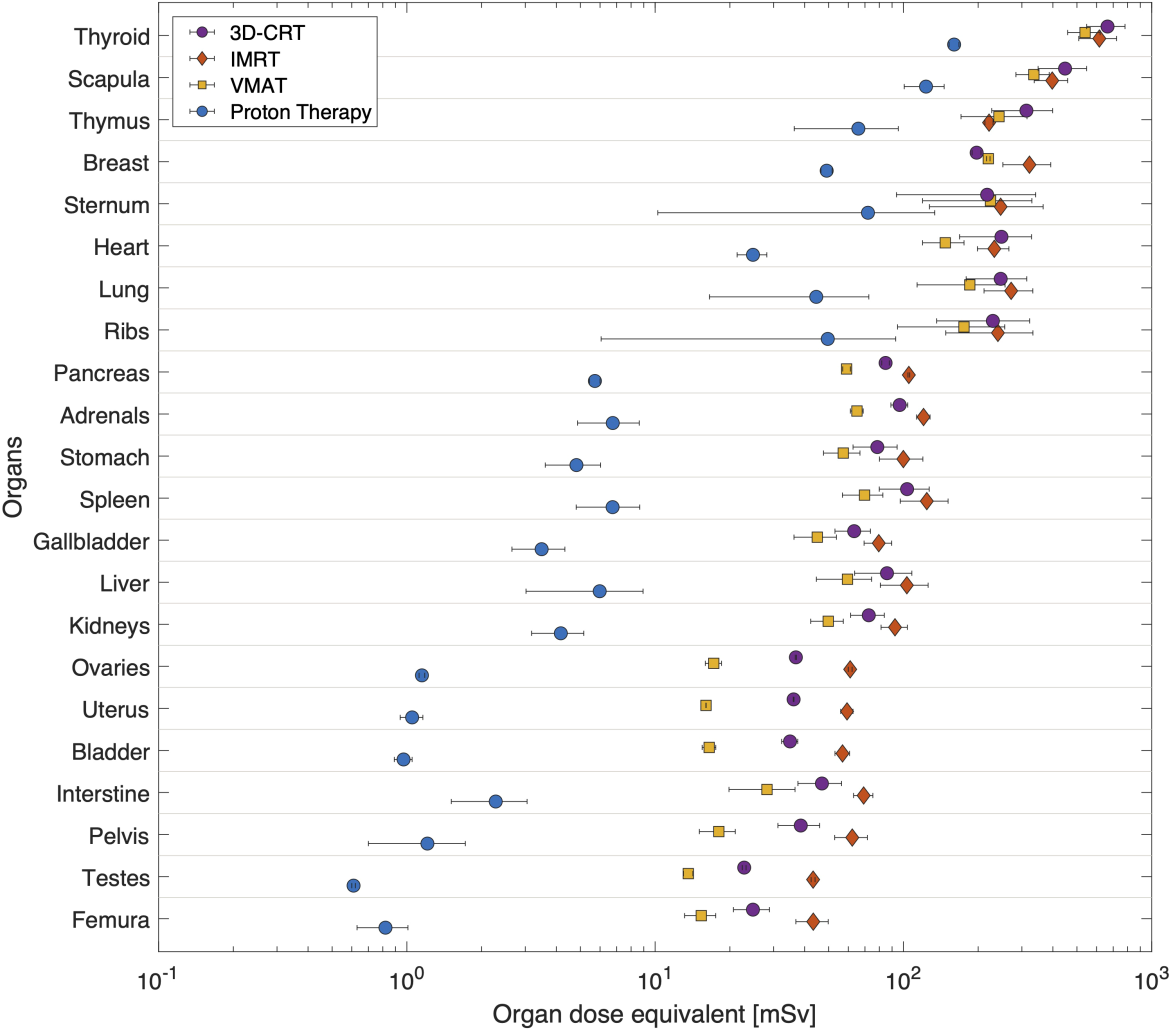
Average dose equivalent per organ from radiotherapy for different techniques, 3D-CRT, IMRT, VMAT and PBS proton therapy. Organs are sorted according to their distance to target. Horizontal bars correspond to the spread of doses as calculated at various locations inside the organ.

In **Figure 3** the out-of-field dose equivalent per organ is plotted for various photon radiotherapy techniques (3D-CRT, IMRT and VMAT) and PBS proton therapy. Within the photon radiotherapy techniques the dose equivalent in thyroid ranges between 500mSv and 620mSv for VMAT and 3D-CRT, respectively. In breast, the dose equivalent is most pronounced for IMRT, 290mSv, as compared to 160mSv and 190mSv for 3D-CRT and VMAT, respectively. For organs in the thorax region, such as lungs and heart, the dose equivalent is more comparable between the different photon techniques. Still VMAT irradiation resulted in lower average lung and heart dose equivalent of 160mSv and 130mSv. The further away from the target, the more visible is the decreased out-of-field dose equivalent for VMAT, when compared to IMRT which yields the highest out-of-field dose equivalent.

The out-of-field dose equivalent during proton therapy is in all cases lower than photon therapy techniques and ranges from 120mSv in the thyroid down to 0.6mSv in testes. The difference to photon techniques becomes larger, the further away from the target. For example, the out-of-field dose equivalent ratio between IMRT and proton therapy ranges from 4.8 in thyroid up to 74 in testes.

### 3.2 Out-of-field dose equivalent per organ from imaging

**Figure 4:**
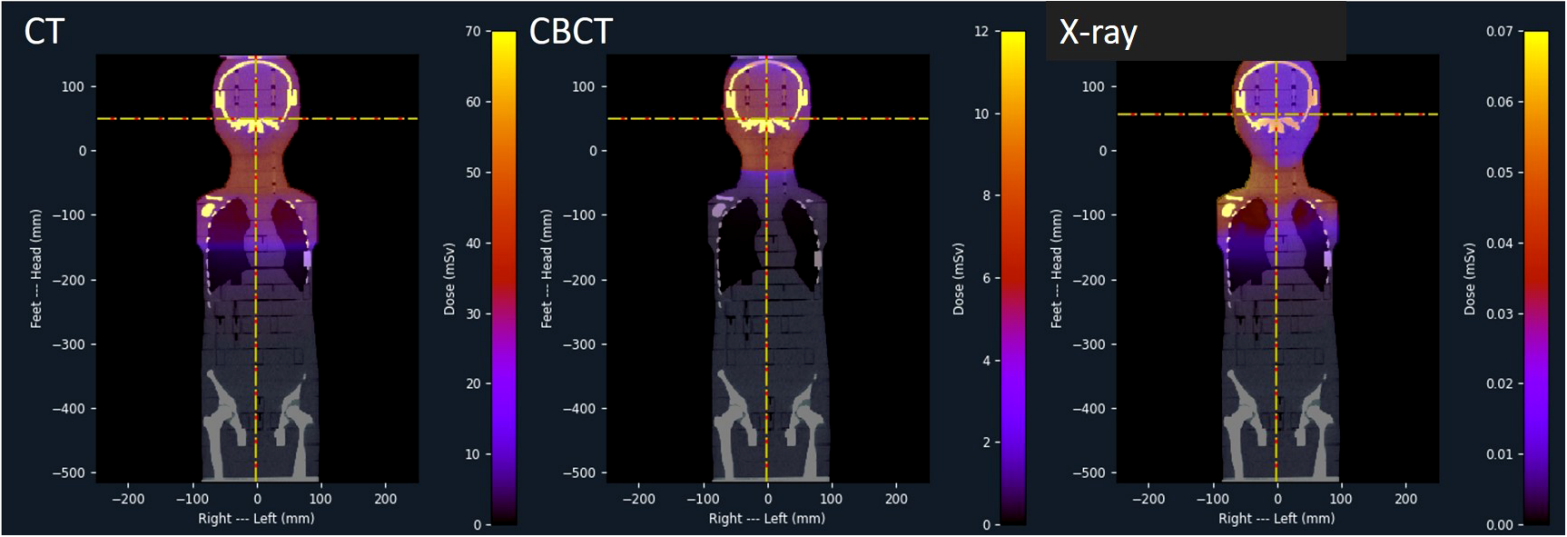
Dose equivalent distributions of CT, kV-CBTC and X-ray as projected on the central coronal plane of the 5-year-old CIRS phantom.

Doses during imaging procedures were calculated using Monte Carlo-based software. **Figure 4** shows the dose equivalent distributions (mSv) projected on the central coronal plane of the 5-year-old CIRS phantom CT. It should be noted that the colour bar scale in that figure is relative to the respective maximum of each modality, namely for CT 0-70mSv, for CBCT 0-12mSv and for X-ray 0-70_μ_Sv.

**Figure 5:**
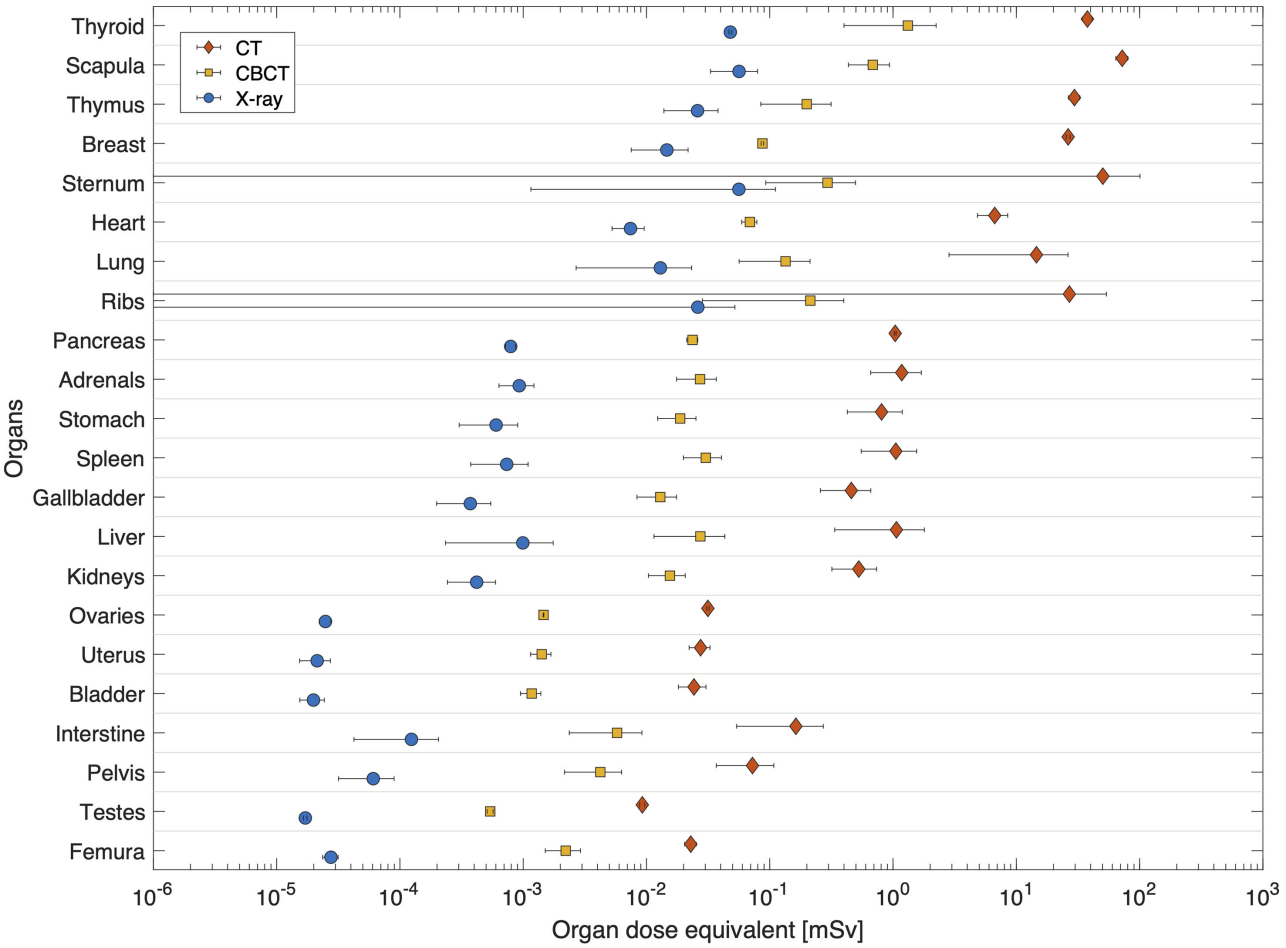
Average dose equivalent per organ for single imaging procedures using a: CT (left), kVCBCT (middle) and X-ray (right). Organs are sorted according to their distance to target. Horizontal bars correspond the spread of dose equivalent as calculated at various plugs inside the organ.

In **Figure 5** the dose equivalent per organ is shown as computed for a single imaging procedure. It is clear that CT results in elevated dose equivalent per organ when compared to OBI techniques such as kV-CBTC and X-ray. CT doses range between 0.01mSv (testes) and 72mSv (scapula) while for CBCT this is between 0.5_μ_Sv (testes) and 1.3mSv (thyroid). For X-ray the dose equivalent ranges between 0.02_μ_Sv (testes) and 56_μ_Sv (scapula). Organs in the thorax region spreading over long distances in the coronal plane, such as sternum, ribs and lungs, demonstrate large spread of computed dose equivalent, indicating a large dose gradient.

**Figure 6:**
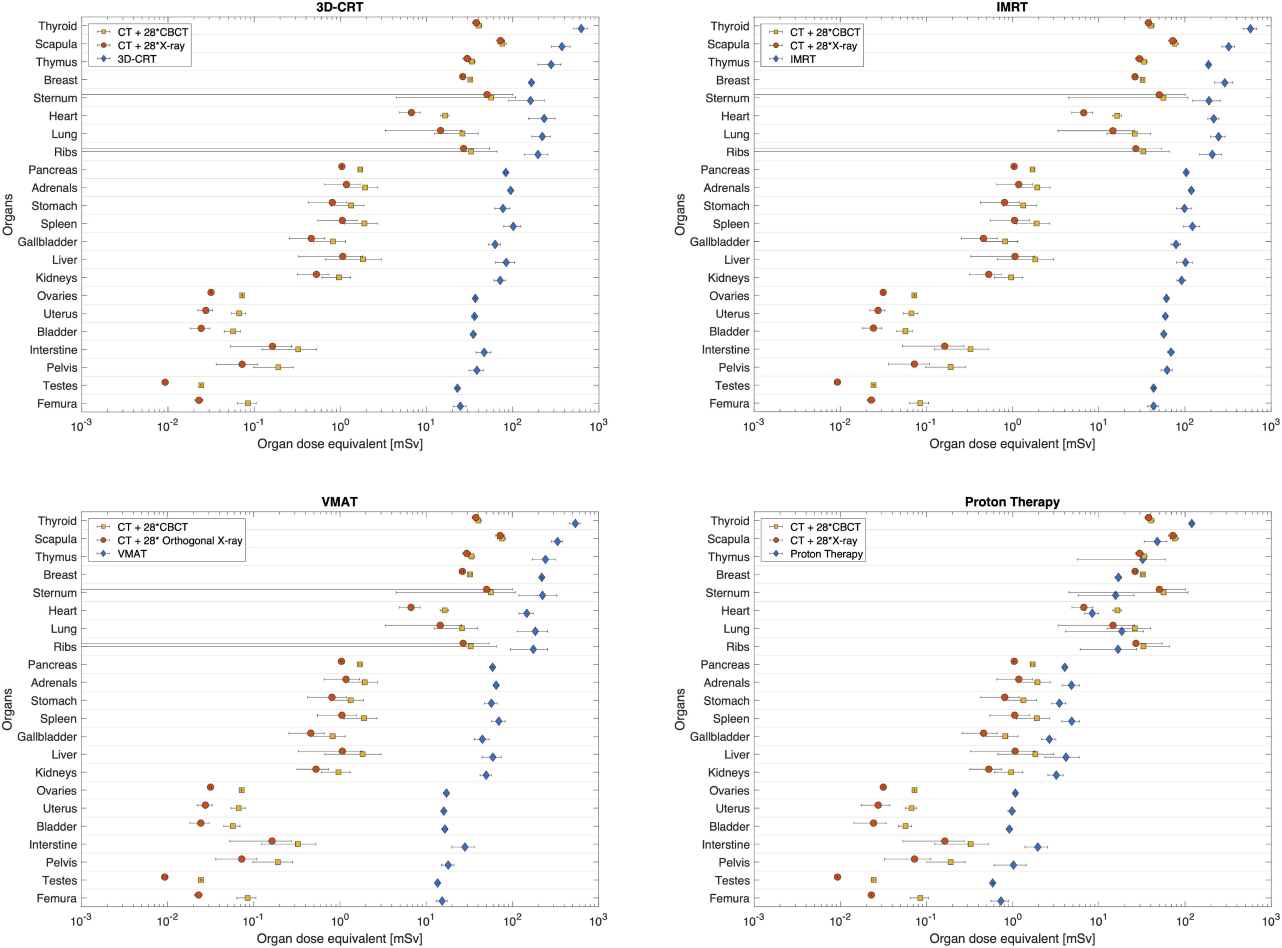
Comparison between total imaging dose equivalent for both, daily CBCT (CT+28*CBCT) and daily X-ray (CT+28*X-ray), as compared to 3D-CRT, IMRT, VMAT and proton PBS therapy. Organs are sorted according to their distance to target. Horizontal bars correspond the spread of dose equivalent as computed at various plugs inside each organ.

When considering a daily imaging procedure, the total imaging dose equivalent per organ is plotted in **Figure 6**. In general, the difference between CT+28*CBCT and CT+28*X-ray is low, as contribution from daily CBCT or X-ray is small when compared to the large contribution from CT.

### 3.3 Comparison between therapeutic and imaging dose equivalent per organ

For photon techniques the imaging dose equivalent is lower than the therapeutic dose equivalent. Still, close to the field, the contribution of imaging dose equivalent can be important (**Figure 6**). For example, for the scapula the imaging dose equivalent for daily CBCT imaging is 29%, 24% and 24% of the dose equivalent during 3D-CRT, IMRT and VMAT, respectively. When comparing imaging dose equivalent per organ to proton therapy, data become more comparable due to the lower out-of-field therapeutic dose equivalent during proton therapy. In organs close to the field the imaging dose equivalent even exceeds the therapeutic dose. For example, in the scapula, the imaging dose equivalent is 59% and 52% higher, respectively, for daily CBCT and X-ray imaging, compared with the proton therapeutic dose equivalent. The largest ratios between imaging dose equivalent and proton therapeutic dose are obtained in the sternum, with ratios of 3.6 and 3.2 for daily CBCT and X-ray imaging, respectively. In the abdomen region, the dose equivalent from imaging becomes smaller than the therapeutic dose during proton therapy as it can be seen for pancreas and other organs further away from the target.

### 3.4 Total dose equivalent per organ and comparison between radiotherapy techniques

**Figure 7:**
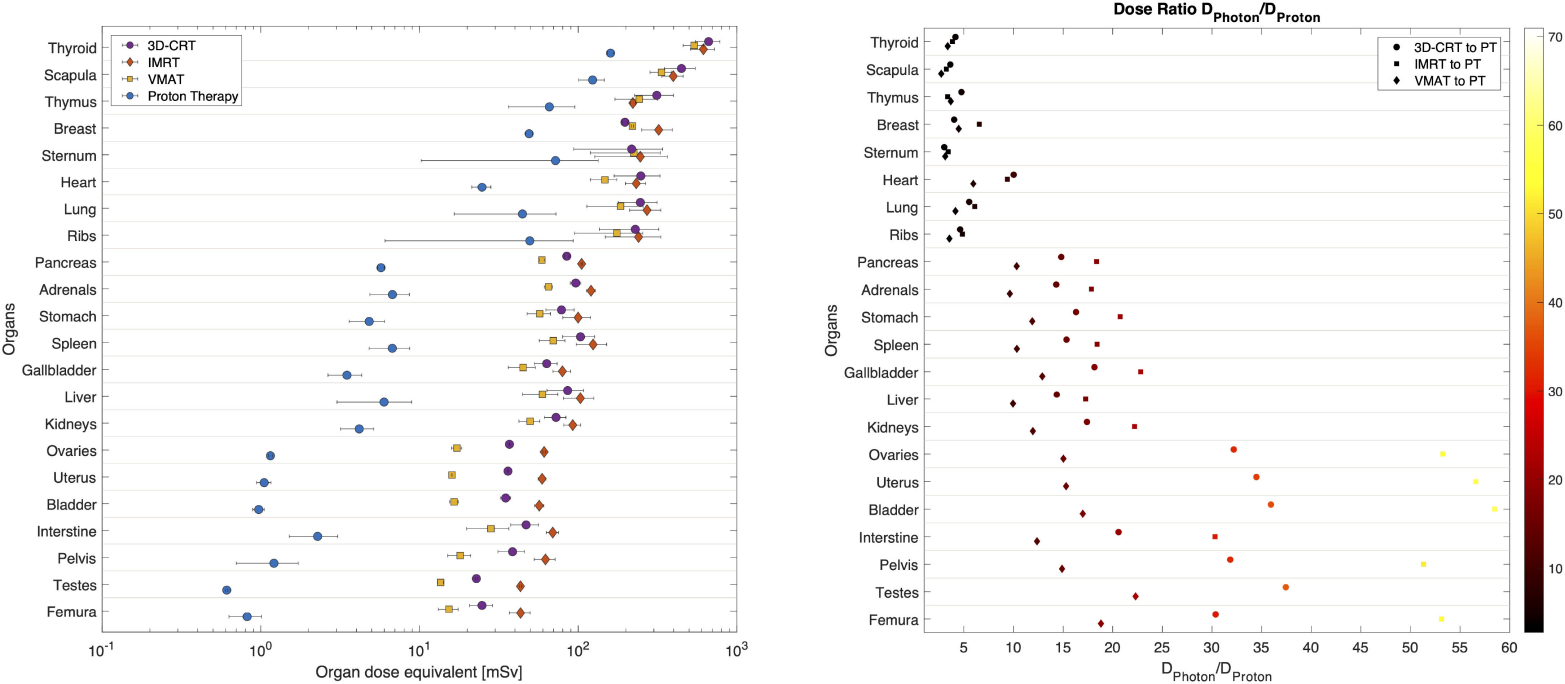
Total dose equivalent per organ calculated for 3D-CRT, IMRT, VMAT and PBS proton therapy, considering a daily CBCT imaging. On the right the photon (3D-CRT, IMRT and VMAT) to proton dose equivalent ratio per organ and corresponding colour bar scale. Organs are sorted according to their distance to target. Horizontal bars correspond to the combined spread of dose equivalent as estimated at various plugs inside each organ.

A final comparison between radiotherapy techniques is made by considering the additional dose equivalent from imaging. Here we use the daily CBCT as the most conservative approach, as it resulted in the most elevated imaging dose equivalent, and compare the total dose equivalent for the different radiotherapy techniques in **Figure 7**. Even when considering the contribution from imaging to the out-of-field dose equivalent during PBS proton therapy, the dose equivalent per organ is significantly lower when compared to photon radiotherapy. Within photon radiotherapy techniques, differences between techniques become more visible the further away from the target. Clearly, IMRT yielded the most elevated out-of-field dose equivalent per organ.

The ratio of photon to proton dose equivalent increases when computed further away from the target. In the thyroid the ratio is around 4 for all photon techniques, while for the bladder the ratio is 36, 58 and 17 when comparing 3D-CRT, IMRT and VMAT, respectively to PBS proton therapy.

### 3.5 LAR for secondary cancer

In **Table 1** LAR is shown for a limited number of organs for which dose-response relationships for cancer induction are available. We tabulated the LAR for each radiotherapy technique and imaging procedure individually as well as the summed LAR for the total doses. It is clear that the most pronounced risk is to develop breast cancer, followed by lung cancer and thyroid cancer. Proton therapy has a reduced risk compared with photon radiotherapy techniques, for the computed out-of-field dose distributions, which are a factor of 9, 13, and 9 for or three-dimensional CRT, IMRT and VMAT, respectively. The summed risk for the selected peripheral organs from proton therapy is slightly lower than the risks from imaging, assuming daily CBCT (CT+28*CBCT). This is mostly because the predicted LAR, for breast and lung cancer, is higher for imaging while other organs show a lower predicted LARs for imaging. When combining the risk calculations, from all investigated organs and including risks from radiotherapy and imaging, the predicted summed risk is the largest for IMRT (3.6%) followed by 3D-CRT (2.6%) and VMAT (2.5%). Proton therapy yields the smallest total LAR (0.6%) which is a factor of 5, 6 and 4 lower when compared to 3D-CRT, IMRT and VMAT, respectively. It must be stressed that these risk estimations are done for peripheral organs and take only into account the risk derived from the respective peripheral absorbed dose distributions obtained from treatment and therapeutic imaging. The summed risk for the considered peripheral organs will be hereafter referred to as ‘partial risk’.

**Table 1:** LAR for selected peripheral organs and summed LAR for those organs (partial risk).

LAR (%)					
	3D-CRT	IMRT	VMAT	Proton Therapy	Imaging
Bladder	0.08	0.12	0.04	0.002	0.000
Breast	1.18	2.04	1.34	0.124	0.236
Liver	0.10	0.11	0.07	0.005	0.002
Lungs	0.45	0.50	0.33	0.039	0.055
Stomach	0.14	0.18	0.10	0.006	0.002
Thyroid	0.37	0.34	0.30	0.076	0.026
Partial risk	2.32	3.30	2.17	0.25	0.32
	3D-CRT + Imaging	IMRT + Imaging	VMAT + Imaging	Proton Therapy + Imaging	
Bladder	0.08	0.12	0.04	0.002	
Breast	1.41	2.26	1.56	0.358	
Liver	0.10	0.12	0.07	0.007	
Lungs	0.50	0.55	0.38	0.094	
Stomach	0.14	0.18	0.11	0.009	
Thyroid	0.39	0.36	0.32	0.100	
Partial risk	2.62	3.60	2.48	0.57	

LAR is computed for each radiotherapy technique and imaging procedure separately, as well as for the total doses of combined radiotherapy imaging procedures.

## 4 Discussion

The current Monte Carlo-based framework allows to study the complete patient exposure during paediatric brain cancer and the potential subsequent risks for secondary cancer induction from available dose-response models. The accuracy of the presented simulations was experimentally shown in previous publications.

Stray radiation in proton therapy is dominated by neutrons, therefore we considered the higher RBE, by applying the Q-factor of neutrons in the Monte Carlo software and reported on dose equivalent per organ, as described in De Saint-Hubert et al. (25). For photon therapy as well as imaging doses we considered the radiation type and assumed a RBE=/Q-factor=1. In this way a fair comparison between the techniques is allowed as radiation type is considered. These results demonstrate dose equivalent per organ were between a few, up to around hundred mSv for proton therapy, while photon techniques are ranging between few tens up to few hundreds of mSv. Furthermore, to allow the comparison between the radiotherapy techniques we featured the same cranial tumour location, size and shape. Still, the treatment plans were established according to the protocols of the individual radiotherapy clinics. These protocols differed regarding the requirements for PTV coverage (see section 3.1), which caused small dose deviations within the PTV. For instance, the median doses of the PTV exhibited differences of up to 2.7%. If these deviations are regarded as uncertainties, the impact on the overall uncertainties is negligible.

In the study from Knežević et al. (32), the comparison between photon therapy, namely 3D-CRT, IMRT and GammaKnife, and PBS proton therapy for brain, revealed a reduction in out-of-field dose equivalent which was at the level of one order of magnitude close to the brain and more than two orders of magnitude further away from the target. Our study showed a similar benefit of proton therapy further away from the field, up to a factor of 58 for bladder when IMRT was compared to proton therapy. Nevertheless, we did not observe differences of more than two orders of magnitude, which could be due to several reasons. First, the present study reported on dose equivalent per organ resulting from a larger target volume (195.2cm^3^) compared to the previous study [65cm^3^ (19, 32)]. Another previous study has shown the impact of clinical plan parameters on ambient neutron dose equivalent, H*(10), in PBS proton therapy as a function of treatment plan parameters. The linear increase with field size and an increase of up to a factor of 8 with an augmenting range were found to be the strongest influences on H*(10) (52). Secondly, the present study used range shifters during PBS proton treatment while this was not the case in the work of Knežević et al. (32). Indeed, it has been demonstrated that the use of a range shifter can increase the out-of-field dose up to more than a factor of 2 (19, 53). Finally, the contribution of imaging procedures was not considered in any previous study. Indeed, we noticed a more significant relative contribution, of the dose equivalent per organ, from imaging when proton PBS therapy is applied when compared to photon therapy. Another reason could be the fact that in the comparative study of Knežević et al., 3D-CRT was done using dynamic and mechanical wedges increasing the out-of-field doses for this technique and hence the ratio of photon to proton dose. Knežević et al. reported lowest out-of-field dose equivalent for IMRT when compared to 3D-CRT and GammaKnife, which may be explained by the relatively low number of monitoring units (209MU) and the use of wedges during 3D-CRT. Herein, IMRT was performed with 682MU and it should be noted that intensity modulation affects the out-of-field dose equivalent in two ways. First, the collimator scatter is increased by a factor roughly proportional to the increase in monitor units. Secondly, due to better conformality, patient scatter is decreased. The higher out-of-field dose for IMRT when compared to 3D-CRT, suggests that the MU increase from 3D-CRT to IMRT is greater than the reduction in patient scatter due to better conformality. Therefore, our work shows an increased risk of secondary cancer induction for IMRT when compared to 3D-CRT. For VMAT (421MU), on the other hand, monitor units do not increase as much as with IMRT and, therefore, the advantage of better conformality (less patient scatter) prevails and the risk for secondary cancer is below that of 3D-CRT.

One needs to be cautious when favouring one technique to another as this comparison is only for the specific out-of-field organs considered for which measurements were performed. Indeed, the published cancer risks represent only an under-estimation of the probable overall risk of secondary cancer, which should include sarcoma, non-malignant brain tumours (e.g. meningioma), carcinoma for organs located in-field and haematopoetic tumours for the overall risk ratio. It must also be pointed out that the cancer risks for organs in the medium and high dose range can behave quite differently with regard to the various irradiation techniques than in the low dose range (54, 55). This is because the dose distribution of the primary radiation is more or less independent of the dose deposition by scattered radiation (which is responsible for the peripheral dose deposition). Therefore, one cannot infer the overall cancer risk from a comparison of the risks of different irradiation techniques for peripheral organs. The cancer risk presentation should be understood as an example of quantitative risk assessment from dose data. One goal of the HARMONIC project is to assess second cancer risk in relation to out-of-field organ doses, with the aim of improving such risk models.

Imaging dose equivalent was most pronounced for CT and more than one order of magnitude higher when compared to CBCT. CT dose equivalent data were, however, higher when compared to the data obtained within the EPI-CT study (56). For example, in EPI-CT the thyroid dose equivalent to a 5-year-old CT scan of the brain was around 10mSv, while in our study it was 38mSv. The higher dose equivalent observed in the presented CT exam may be due to several factors. First, the scan covered a larger section of the patient’s body compared to the EPI-CT study, where thyroid was out-of-field. Scan length was shown to play a crucial role and effective dose was increased as a function of length with 15%/cm on average (57). Additionally, EPI-CT being a radiology study, protocols may be better adapted to the patient morphology. In contrast, planning CT scanners typically use fewer protocols often relying on a single kVp setting. This led, in our case, to the use of 120kVp, even for head and paediatric exams where lower voltage settings would have been preferred in diagnostic radiology. Moreover, the protocol used did not apply current modulation techniques to reduce radiation exposure and spare dose in thin regions of the patient’s body, such as the neck region.

CBCT yielded dose equivalent data that were lower when compared to previously published data (58). Our study calculated doses between one mSv, close to the field and less than a _μ_Sv at far distances while in the study from Hälg et al. (58) kV-CBCT dose data from different manufacturers, range between an average dose around 10mGy at 10cm and 0.1mGy at 50cm from the isocentre. Although there may appear to be discrepancies, the reported doses are actually compatible. In fact, our ‘head low dose’ protocol is similar to the ‘high quality head’ protocol (59), with the main distinction being that the high-quality protocol uses 5 times more mAs (due to different image quality target), which directly translates to delivering 5 times more radiation dose to the patient. Moreover, reported organ doses cannot be easily compared directly since the treatment site and the region in the two studies are different.

The dose equivalent from X-ray imaging was more than one order of magnitude lower than kV-CBCT. We would like to note that during the first treatment session, the ‘Kopf Kind G0A’ protocol is repeated one additional time at each one of the three gantry angles used by the proton treatment (70°, 110°, 260°). The extra X-ray procedures are only done at the first radiotherapy treatment but we have calculated the impact of this extra dose. As expected the dose equivalent from X-ray procedure increased and this was on average by a factor of 3, when compared to a single angle (gantry 0°). However, this was only for the first treatment fraction and the impact on the total dose equivalent was within 15%. Therefore, we did not report on the extra dose from different angles. Moreover, X-ray doses are so low that the contribution to the total dose equivalent will be very limited. For this reason, the total dose equivalent applied during radiotherapy was calculated for daily OBI with kV-CBCT, which would result in the most conservative estimate of the dose equivalent per organ and associated risk.

Typically, bone structures receive higher doses than soft tissues at similar distances from the field. This is expected due to the energy range of photons used for imaging and the resulting higher mass attenuation coefficient of bones compared to soft tissues. Additionally, dose spread appears to be greater in bones. High dose gradients are particularly noticeable in organs such as the sternum, lungs, and ribs for CT and X-ray. In these cases, the extreme dose spread could be attributed to the fact that, for both CT and X-ray, the dose fall-off is located in the lung region, while for CBCT, it is in the neck area due to its smaller imaging field of view measuring only 17cm along the patient’s length, compared to approximately 30cm for the other modalities. Overall, the importance of imaging dose is highlighted in our study (60) and strengthens the necessity to increase awareness on CT procedures (61, 62) as well as on-board imaging in this specific application, namely radiotherapy in paediatric populations (63, 64). The relative contribution from imaging to the total dose equivalent per organ is more pronounced for proton therapy when compared to photon therapy techniques. This is also reflected in the associated risks, demonstrating a similar risk from imaging and therapeutic exposure. Risk of second cancers for far out-of-field organs may account for less than 20% of all second cancers developed (even though this proportion depends on the follow-up time and attained aged considered) (65). The computed risk of secondary cancer following 3D-CRT, IMRT, VMAT and PBS proton therapy are, respectively, 2.6%, 3.6%, 2.5% and 0.6%, which is in line with the study from Xiang et al. (10) that also suggests a lower risk for secondary cancer when using protons, while IMRT and 3D-CRT showed similar risks. More specifically, for primary tumours of the head and neck, proton therapy was associated with a significantly lower risk for secondary cancer (adjusted [OR], 0.42; 95% CI, 0.22–0.81; *P* = 0.009). In our study the risk was reduced by a factor of 6 when studying protons versus IMRT, which could be related to the fact that we did not calculate the risk to all organs, because of missing dose-response relationship for some organs, as well as the fact that we considered only organs far out-of-field. Moreover, the study of Xiang et al. (10) showed a modest decreased risk of secondary cancer for head and neck cancer treated with IMRT when compared to 3D-CRT (adjusted [OR], 0.85; 95% CI, 0.77–0.94; *P* = 0.001). This was not observed in the current study, where the risk estimations show a reduced risk for 3D-CRT compared to IMRT. One possible explanation is that this work only analyses the cancer risk for organs in the low-dose volume. However, in the low dose volume, the increased scatter and leakage dose with IMRT contributes to an increased cancer risk for these organs. For organs that are in the high-dose range and not included in this study, IMRT reduces cancer risk because of the higher conformality relative to 3D-CRT. Moreover, it should be noted that the study of Xiang et al. (10) was based on a short follow-up time when considering secondary cancer, especially those which may arise in the low dose region.

The results of the present study should be considered under certain limitations. First, our results are specific for the type of brain cancer studied and cannot be directly applied to other malignancies. Secondly, the calculated doses are based on a CIRS phantom and, thus obtained for the given geometry and material composition of this phantom. CIRS has developed materials that mimic the linear attenuation curves of real tissue but the material composition is, of course, different from actual tissue. In the case of proton therapy, in which the out-of-field dose is dominated by secondary neutrons, the material composition may impact on the obtained doses. Third, the organ dosimetry is calculated under certain assumptions such as setting the RBE=/Q-factor=1 for photon radiotherapy and all imaging procedures, as well as summing the organ doses from the different procedures to get an overall dose equivalent per organ. The latter is open for debate, but no other methods have been described so far. Furthermore, the average organ doses are compared as calculated based on point measurements within the organ and do not allow to compare organ dose distributions or dose-volume histograms (DVH). Even though a simple analytical model for a fast 3D assessment of out-of-field doses has been proposed for photon radiotherapy (66), the DVHs would not alter our findings due to the small dose gradient in the out-of-field organs. Finally, the most important limitation is likely to be the risk model employed, which is based on epidemiological studies from A-bomb survivors and Hodgkin’s lymphoma adult patients. It is known that the accuracy of the predictions of this model is limited, however, to the best of our knowledge, this is one of the most adequate models currently available. Dedicated epidemiological studies on paediatric cohorts with modern radiotherapy techniques are required. The HARMONIC project is building a European registry of children and adolescents treated with modern radiotherapy techniques, which contains DICOM files, in addition to clinical, biological and follow-up data. This database will effectively open the possibility to future epidemiological studies to, in turn, improve current risk models.

## 5 Conclusion

In this study we demonstrated the use of a validated Monte Carlo framework calculating the complete dose equivalent per organ, including the therapeutic and imaging procedures. We reported on the complete patient exposure during paediatric brain cancer treatment, showing a significant contribution from imaging to the out-of-field dose equivalent per organ when proton therapy is used, due to the lower dose equivalent from proton therapy compared to photon therapy techniques. For the specific out-of-field organs studied, it was shown that proton therapy decreases the out-of-field doses and associated risk for secondary cancer.

## Data availability statement

The raw data supporting the conclusions of this article will be made available by the authors, without undue reservation.

## Author contributions

MD-H: dose and risk calculations, data analysis, writing. GB: imaging dose simulations, writing. US: treatment-planning, risk calculations, data analysis, writing. CB: treatment planning, imaging protocols, writing-review. NV: proton simulations, writing-reviewing. JE: Monte Carlo geometry coding. JW: proton simulations. FSt: photon simulations. FSu: photon simulations and data analysis. RN: data analysis. JD: methodology, writing-review. FV: data analysis. SR: data analysis. MR: photon simulations, data processing. AS: CT scanner geometry, NJ: epidemiological analysis, writing-review. BT: clinical analysis, writing-review. IT-C: project coordinator, writing-review. LB: Monte Carlo simulations, conceptualization, supervision, writing, writing-review, editing. All authors contributed to the article and approved the submitted version.
