# Supplementary material for: Complete patient exposure during paediatric brain cancer treatment for photon and proton therapy techniques including imaging procedures
Source: Front Oncol. 2023 Sep 19;13:1222800. doi: 10.3389/fonc.2023.1222800 (PMC10546320; doi:10.3389/fonc.2023.1222800)
Supplement: Supplementary file 1 [file DataSheet_1.pdf]

Supplementary Material

1 MONTE CARLO VALIDATION DATA FOR THE CT SCANNER AND THE ORTHOGONAL IMAGING SYSTEM

Here, we provide validation data for the Monte Carlo model of two imaging systems: the Philips Big Bore scanner and the stereo imaging system installed on the IBA proton gantry at the WPE. To test the accuracy of each model, we conducted dose measurements using optically stimulated luminescence (OSL) detectors (Nanodot from Landauer, Inc., Glenwood, IL) on an anthropomorphic phantom (ATOM, Computerized Imaging 69 Reference Systems (CIRS), Inc., Norfolk, VA) designed to represent a five-year-old child (type 705D).

For every native beam quality used at the proton therapy center, OSL detectors were first calibrated in air kerma against a farmer-type ionization chamber (0.6 cc, PTW30013). The OSL measurements taken inside the phantom were then converted into dose to medium by applying energy absorption coefficient ratios (weighted by beam quality) between the calibration medium (air) and the measurement medium (anthropomorphic phantom materials) (Boissonnat et al., 2023).

In both scenarios, Monte Carlo calculations were conducted to replicate the respective examinations using the DICOM file generated by the CT procedure as the reference geometry. Due to the possibility of slight movements of OSLs within their inserts when positioned in the anthropomorphic phantom, their precise location in the CT image cannot be accurately determined. Instead, a sphere with a radius of 5 mm is marked around the assumed location of each OSL, and the doses calculated in each pixel of this sphere are recorded. This generates a dose distribution (rather than a single dose value) for each experimental point. In addition, as our Monte Carlo models are computing only relative doses, a calibration is required for each beam model used.

1.1 Philips Big Bore CT scanner

As a validation protocol, we conducted a full-length imaging of the paediatric phantom using the following parameters: 120 kVp, 12 mm collimation, current modulation activated (with an averaged current of 91 mA), 688 mm of scan length and an exposure time of 52.9 s. OSLs were positioned at 53 positions all along the phantom from the head to the pelvis. In order to increase the signal-to-noise ratio for OSL measurement the exam was repeated four times.

1.2 IBA's gantry mounted orthogonal kV imaging system at WPE

For validation, we utilized the 'head low dose' protocol with the proton gantry placed at 0°. This protocol involves taking two X-ray images: the first using X-ray tube A, in the same direction as the treatment beam at 90 kVp, 12 mA, and 100 ms (with a source-axis distance (SAD) of 1511 mm, field size of 20.2 cm x 27.9 cm); and the second using orthogonal X-ray tube B, oriented at 270° from the treatment beam direction at 90 kVp, 32 mA, and 100 ms (with an SAD of 2870 mm, field size of 24.4 cm $\times$ 33.8 cm).

OSLs were placed at 50 locations along the imaged portion of the phantom, from the head to the heart. To improve the signal-to-noise ratio for OSL measurement, the examination was repeated fifty times.

To calibrate Monte Carlo results into dose, the air kerma at the isocenter was measured and simulated for an irradiation with both X-ray tubes and using a PTW30013 ionization chamber placed inside an 18 mm thick PMMA plate.

1.3 Validation results and discussion

In the case of the Big Bore CT scanner (Figure S1), the simulated and measured data show good agreement, following the trend of CTDIvol values within a factor of two for soft tissues (and a factor of four for bony tissues), as expected because the displayed CTDIvol value by the scanner is estimated for a CTDI phantom of 32 cm, which is more representative of an adult morphology.

The relative deviations highlight a systematic deviation of 21% and a dispersion of $\pm15\%$. While this level of dispersion was expected based on the assumptions made in the model construction (constant incident beam quality over the entire scanner aperture, scanner table description) and conversion to dose in the medium for OSL (conversion to dose in the medium based on incident beam quality), the systematic deviation of 21% is more surprising and should be the subject of further study. It could be explained by the use of a PTW 0013 ionization chamber for the simulation calibration, as this ionization chamber measures 24 mm in length while the collimation used for this examination is 12 mm. Therefore, any slight error in the positioning or modeling of the collimation system might influence the estimated quantities.

In the case of positional imaging exam (Figure S2), there is generally good agreement with doses lower than 0.05 mGy for soft tissues and mostly between 0.05 and 0.15 mGy for bones. If the average difference between measured and simulated doses is around 2%, it is noted that the dispersion falls within a range of $\pm25\%$. However, this difference tends to be widened by points corresponding to bone tissues, which also exhibit the highest dose dispersions within a 5 mm radius sphere. These dose dispersions within the spheres can be explained by the fact that the beam only passes through two incidences. Indeed, while dose profiles along a single beam tend to show the highest gradients in high-density regions, these gradients tend to be smoothed out in CT or CBCT dose maps that utilize multiple beam incidences.

REFERENCES

Boissonnat, G., Morichau-Beauchant, P., Reshef, A., Villa, C., Désauté, P., and Simon, A.-C. (2023). Performance of automatic exposure control on dose and image quality: comparison between slot-scanning and flat-panel digital radiography systems. *Med Phys* 50, 1162–1184

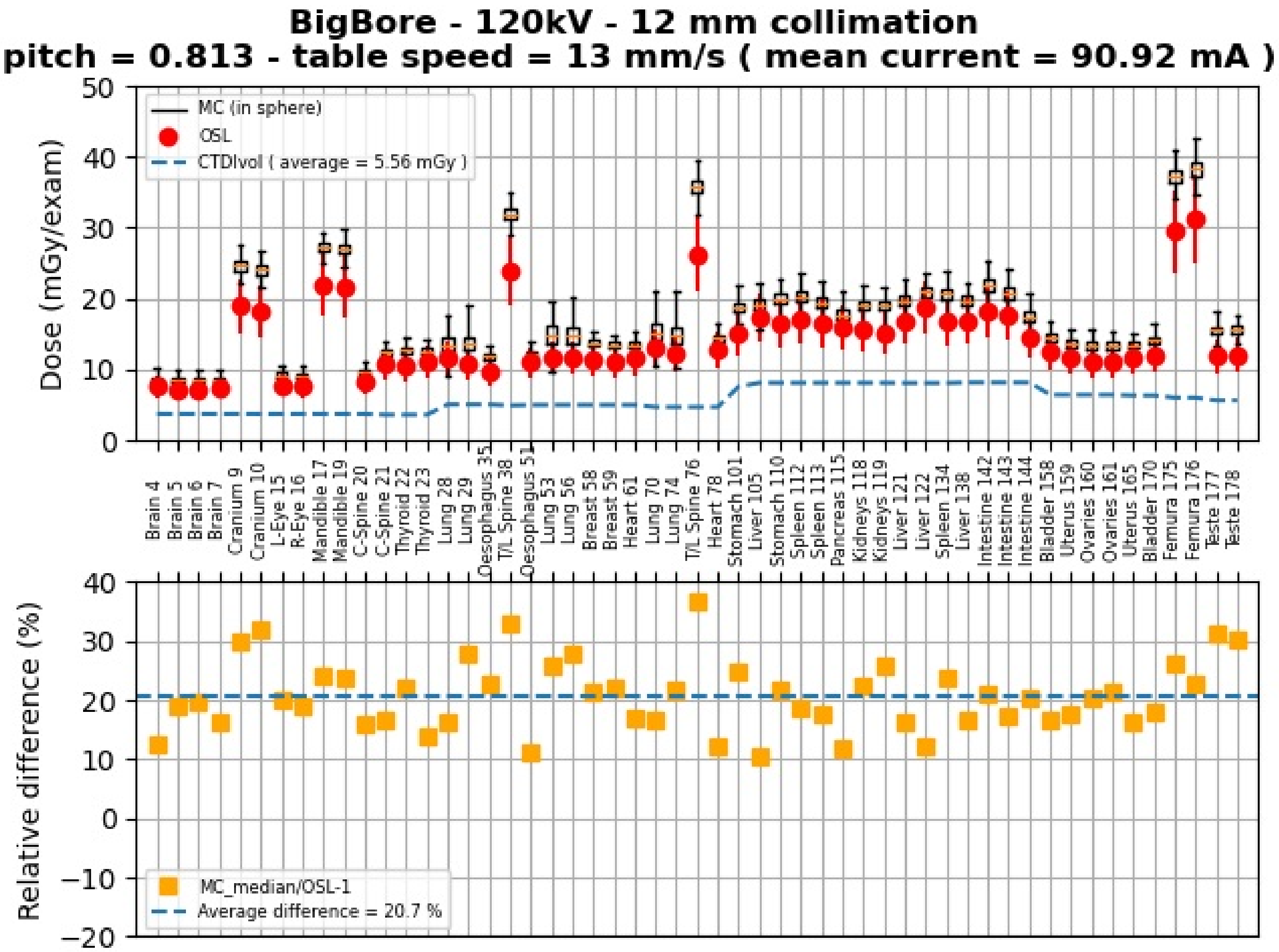

Figure S1. Comparison between measured and simulated doses in medium for a full body exam performed on a 5-year-old paediatric phantom (CIRS/ATOM) using a Big Bore (Philips) CT scanner (120 kVp, 12 mm collimation, modulated current). On top, OSL measurements (red circles) are presented with experimental error bars (extended at k = 2) ; for Monte Carlo data (black squares) each point represents the dose distribution observed in a sphere of 5 mm radius around the assumed position of the OSL ; CTDIvol values from the CT DICOM file are also reported at each measurement location (blue dashed line). On the bottom, relative difference between the doses measured by OSL and the median dose obtained by Monte Carlo (in the 5 mm sphere).

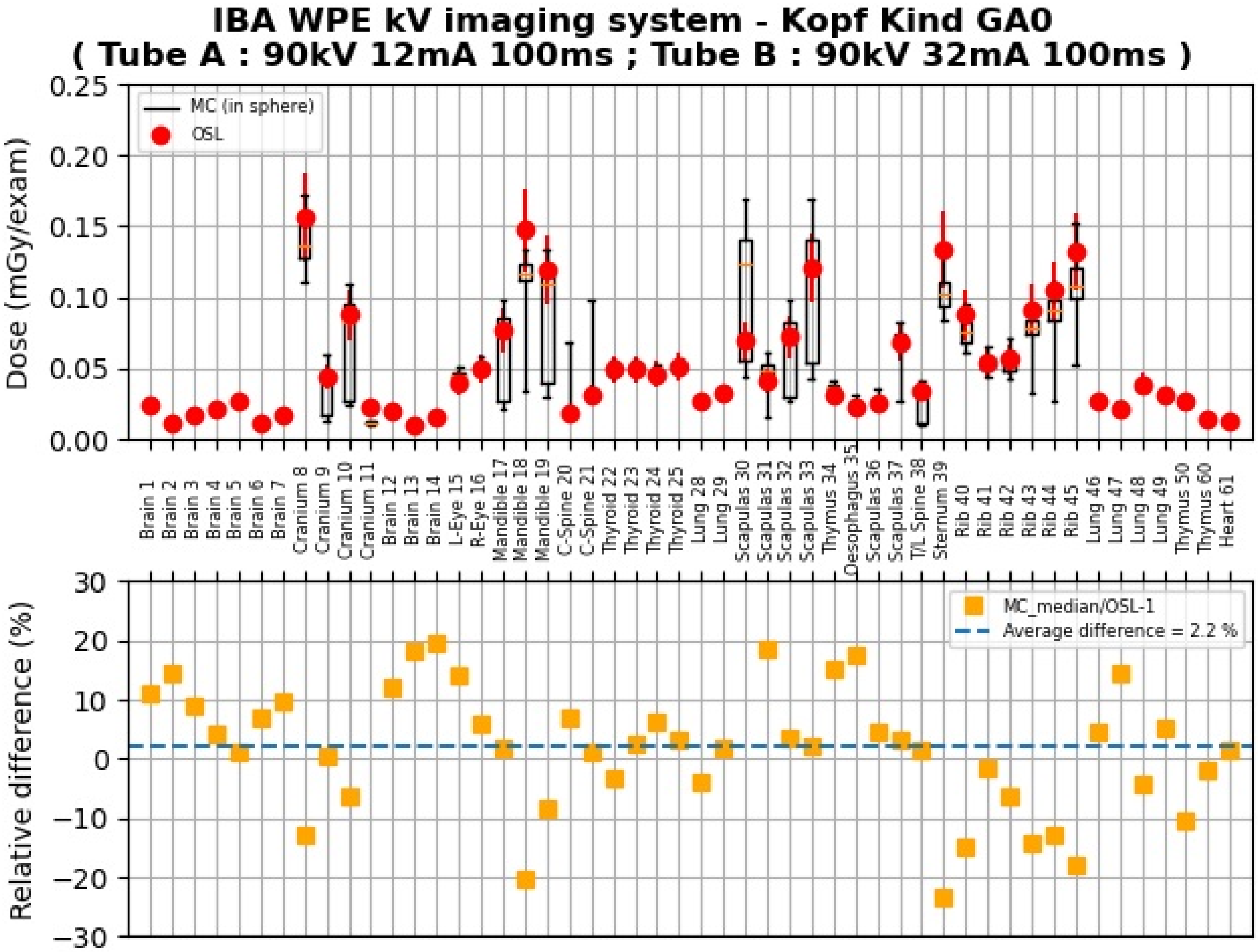

Figure S2. Comparison between measured and simulated doses in medium for a single imaging procedure performed on a 5-year-old paediatric phantom (CIRS/ATOM) using WPE's gantry mounted orthogonal kV imaging system (IBA) 'Kopf King GA0' protocol (Tube A : 90 kVp, 12 mA, 100 ms; Tube B : 90 kVp, 32 mA, 100 ms). On top, OSL measurements (red circles) are presented with experimental error bars (extended at $k=2$) ; for Monte Carlo data (black squares) each point represents the dose distribution observed in a sphere of 5 mm radius around the assumed position of the OSL. On the bottom, relative difference between the doses measured by OSL and the median dose obtained by Monte Carlo (in the 5 mm sphere).
